# The Korean Pregnancy Outcome Study (KPOS): Study Design and Participants

**DOI:** 10.2188/jea.JE20200055

**Published:** 2021-06-05

**Authors:** Hansol Choi, Dong Wook Kwak, Min Hyoung Kim, Su Young Lee, Jin Hoon Chung, You Jung Han, Hee Jin Park, Moon Young Kim, Dong Hyun Cha, Seul Koo, Joong-Yeon Lim, Hyun Mee Ryu, Hyun-Young Park

**Affiliations:** 1 Department of Epidemiology and Health Index, Center for Genome Science, Korea National Institute of Health, Cheongju, Korea; 2 Department of Obstetrics and Gynecology, Ajou University School of Medicine, Suwon, Korea; 3 Department of Obstetrics and Gynecology, Mizmedi Hospital, Seoul, Korea; 4 Department of Psychiatry, Myongji Hospital, Hanyang University College of Medicine, Goyang, Korea; 5 Department of Obstetrics and Gynecology, Asan Medical Center, Ulsan University Medical School, Seoul, Korea; 6 Department of Obstetrics and Gynecology, CHA Gangnam Medical Center, CHA University, Seoul, Korea; 7 Department of Research Planning, Center for Biomedical Science, Korea National Institute of Health, Cheongju, Korea; 8 Department of Obstetrics and Gynecology, CHA Bundang Medical Center, CHA University, Seongnam, Korea; 9 Center for Genome Science, Korea National Institute of Health, Cheongju, Korea

**Keywords:** cohort, pregnancy, pregnancy complications, women’s health

## Abstract

**Background:**

The Korean Pregnancy Outcome Study (KPOS) was established to investigate the determinants of adverse pregnancy outcomes among Korean women.

**Methods:**

We recruited 4,537 pregnant women between 2013 and 2017 from two tertiary centers located in Seoul, Korea, and a total of 4,195 Korean women met inclusion criteria in the baseline analysis. A range of data on socio-demographics, past medical histories, reproductive information, health-related behaviors, psychological health and clinical information were obtained using interviewer-based questionnaires and clinical assessment at 12, 24, and 36 gestational weeks (GW), delivery and 6–8 weeks postpartum. Blood samplings were performed at 12, 24 and 36 GW, and placental tissues were obtained after delivery. The main outcome of this study was pregnancy-related complications including gestational diabetes mellitus (GDM), gestational hypertension, and screening positive for peripartum depression. Depression was assessed using the Korean version of the Edinburgh Postnatal Depression Scale, and a score of ≥10 indicated a positive screen for depression.

**Results:**

Among 4,195 eligible pregnant women with a median age of 33.0 years, 3,565 (85.0%) pregnancy outcomes were available in this study, including 30 miscarriages, 16 stillbirths, and 3,519 deliveries. Mean gestational age was 38.8 GW, and mean birth weight was 3,236 gram. The prevalence of pregnancy complications of GDM, hypertensive disorders, and screening positive of depression during pregnancy and postpartum was 7.0%, 1.4%, 27.8%, and 16.6%, respectively.

**Conclusions:**

We designed KPOS to identify the determinants of pregnancy-related outcomes, and it may provide effective strategies for the prevention of pregnancy complications in Korean pregnant women.

## BACKGROUND

South Korea (hereafter, Korea) has experienced a rapid change in reproduction. It has gone from being a high-reproductive society to one with the lowest of reproductive rates.^1^^–^^3^ Several studies have reported that the low reproductive rate is mainly attributable to delays in marriage and childbearing.^2^^,^^4^^–^^6^ Especially in Korea, the average age of women at the time of marriage has increased consistently over the past decades because of rapid economic growth and notable increase in rates of education, which have paralleled the rise in the childbearing age.^2^^,^^5^^,^^7^ Between 1995 and 2016, the average age of women at first childbirth rapidly increased from 26.5 to 31.4 years among Korean women, whereas the Organisation for Economic Co-operation and Development average was 28.9 years old in 2016.^8^ The increase in the number of *in vitro* fertilizations with new developments in artificial reproductive technologies also contributed to the high rate of women conceiving at an advanced maternal age in Korea.^7^^,^^9^

It is well-established that advanced maternal age is associated with potentially adverse maternal and foetal outcomes.^9^^–^^22^ Older pregnant women are at higher risk of having an adverse pregnancy-related outcome compared to younger women.^9^^–^^14^ Current evidence suggests a strong association between advanced maternal age and adverse obstetric outcomes, including chromosomal abnormalities, stillbirths, miscarriages, congenital anomalies, and perinatal deaths.^12^^,^^15^^–^^19^ Moreover, pregnancy induced complications, such as hypertensive disorders of pregnancy or gestational diabetes mellitus (GDM), may have lifelong adverse health outcomes for the pregnant women and their child, and they can lead to enormous burdens on families, communities, and society as a whole.^9^^,^^23^^–^^25^ However, there has been no well-organized study for the evaluation of pregnancy complications and perinatal outcomes in Korean pregnant women. Several authors reported adverse pregnancy outcomes in high-risk patients and related risk factors, but causal relationships remain undetermined because of the lack of prospective studies.^26^^–^^29^ Therefore, we developed the Korean Pregnancy Outcome Study (KPOS), which is a prospective study on general pregnant women in Korea, aiming to investigate the risk factors and distribution of adverse pregnancy outcomes, specifically GDM, hypertensive disorders of pregnancy, and screening positive for peripartum depression (during pregnancy or at 4 weeks postpartum). In addition, KPOS is aimed to determine the effect of well-known risk factors (ie, anemia, hyperemesis, obesity, and excessive or suboptimal weight gain) and beneficial effect of health-related behaviour (ie, iron or multiple micronutrient supplementation and physical activity) to pregnancy complications and neonatal outcomes.

The aim of this article is to describe the design of the KPOS, present the characteristics of the study populations, and briefly report the results of the study.

## METHODS

### Study participants

**Figure 1.**
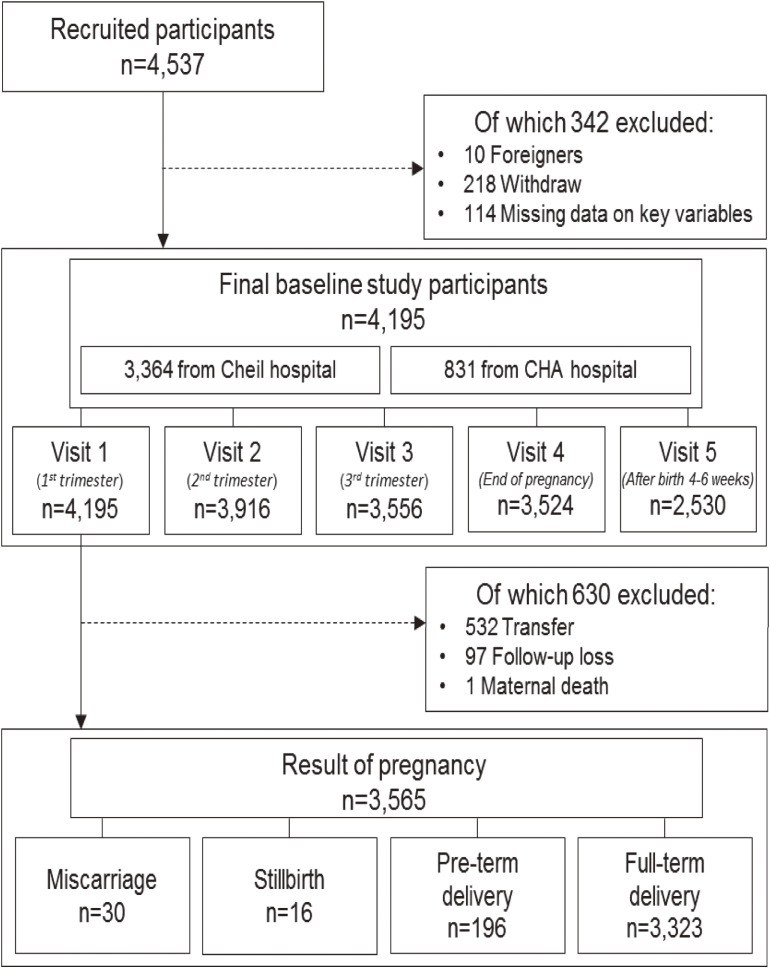
The flowchart of KPOS participants.

Between March 2013 and January 2017, all pregnant women who visited Cheil General Hospital & Women’s Healthcare Center and CHA Gangnam Medical Center for antenatal care during the first trimester were asked to participate in the KPOS. Trained research nurses explained the study in detail and obtained written informed consent from participants and assisted them in completing the interview-based questionnaires. Women were excluded from enrolment if they were not Korean or were pregnant with triplets or higher-order multiple gestations. Figure 1 shows the number of participants recruited and the flow of participants through the KPOS.

During the baseline period, we recruited 4,537 participants. Both Cheil General Hospital and CHA Gangnam Medical Center are general hospital which focuses on obstetrics and gynaecology located in Seoul and approximately 5,000 and 2,500 deliveries, respectively, take place at these facilities per year, respectively. A total of 4,195 pregnant women from Cheil General Hospital (*n* = 3,364, 80.2%) and CHA Gangnam Medical Center (*n* = 831, 19.8%) were included in the baseline analysis after excluding 10 foreigners, 218 individuals who declined to participate in the study, and 114 individuals who were missing key variables. In this study, we have defined ‘foreigners’ as people who are neither Korean citizens nor ethnically Korean. Gestational age was determined by the last menstrual period in naturally conceived women and was confirmed on first trimester ultrasound. If the difference between gestational age by last menstrual period and that by first trimester ultrasound exceeded 7 days, the gestational age according to ultrasound dating was used. In the case of *in vitro* fertilization pregnancies, the gestational age was determined based on the date of oocyte retrieval.

After the first antenatal visit, eligible participants were requested to complete several sets of interview-based questionnaires, and clinical assessment such as measurement of maternal body weight and blood pressure were performed at each visit. Clinically assessed data were transferred from medical record. Table 1 outlines the variables measured in pregnant women in the KPOS at each of the following visits: Visit 1 in the 1st trimester (around 12 weeks of gestation); Visit 2 in the 2nd trimester (around 24 weeks of gestation); Visit 3 in the 3rd trimester (around 36 weeks of gestation); Visit 4 at delivery; Visit 5 at 4–6 weeks postpartum. Of the 4,195 women involved at baseline, 630 were excluded at the time of delivery (attrition rate: 15.0%) because 532 women changed hospitals during the antenatal period, 97 women were lost to follow-up, and one woman died during pregnancy.

**Table 1.** Outline of measures for pregnant women in KPOS research

	***Gestational weeks and trimester of visit***				

	***1st***	***2nd***	***3rd***	***4th***	***5th***
	*(Before 12 weeks)*	*(24 weeks)*	*(36*–*40 weeks)*	*(Delivery, End of pregnancy)*	*(After birth 4*–*6 weeks)*
***Consent form***	✓				
***Socio-demographics***					
Marital status	✓	✓	✓		✓
Type of family	✓	✓	✓		✓
Employment status	✓	✓	✓		✓
Educational status	✓				
Household income	✓				
Occupation	✓	✓	✓		✓
Spouse’s information	✓				
***Medical history***	✓				
History of disease	✓				
Family history of disease	✓				
Medicine treatment history	✓				
***Reproductive information***					
Information of current pregnancy	✓				
Maternal illness during pregnancy	✓	✓	✓	✓	
History of pregnancy	✓				
Pregnancy outcomes				✓	
Breast feeding					✓
***Health-related behaviours***					
Cigarette smoking	✓	✓	✓		✓
Alcohol intake	✓	✓	✓		✓
Physical activity	✓	✓	✓		✓
Sleep duration	✓	✓	✓		✓
Dietary intake	✓				
Health supplement intake	✓	✓	✓		✓
***Psychological health***					
Perceived stress	✓	✓	✓		✓
Satisfaction with marriage	✓	✓	✓		✓
Depression	✓	✓	✓		✓
Quality of life	✓	✓	✓		✓
***Clinical measurements***					
Height	✓			✓	
Weight	✓	✓	✓	✓	
Blood pressure	✓	✓	✓	✓	
Laboratory results	✓	✓	✓		
***Biological specimens***					
Blood	✓	✓	✓		✓ (GDM)
Placenta				✓	

GDM, gestational diabetes mellitus; KPOS, Korean Pregnancy Outcome Study.

### Ethical statement

All participants provided written informed consent, and the study protocol was approved by the Institutional Review Board (IRB) of Cheil General Hospital (IRB number: CGH-IRB-2013-10) and CHA Gangnam Medical Center (IRB number: 2013-14-KNC13-018), separately. It was emphasized to all participants that they were free to withdraw from any part of the study at any point in time.

### Sample size calculation

To evaluate relevance of the required sample size (*n* = 3,000), we estimated the minimum detectable odds ratios of the exposure and the outcome as shown in eTable 1. Power and a two-sided ɑ were set a priori at 80% and 0.05, respectively. The correlation coefficient between main exposure and other covariates was assumed to be 0.2. Prevalences of GDM and peripartum depression in Korea were assumed to be 5% and 10%, separately. Power and a two-sided ɑ were set a priori at 80% and 0.05, respectively. Based on the 33% prevalence of exposure, the minimum detectable odds ratios were estimated to be 1.64 for GDM and 1.45 for peripartum depression. Based on the 25% prevalence of exposure, the minimum detectable odds ratios were estimated to be 1.70 for GDM and 1.49% for peripartum depression. To maintain enough statistical power, we decided to a total of 4,000 participants was anticipated for 3 years. Despite the consecutive recruitment of participants, the enrolment period was inevitably longer than expected with the lowest birth rate in Korea.

### Measurements

#### Socio-demographic profiles

Table 2 shows the details of data and material collection in KPOS. Participants provided their socio-demographic information via interview-based questionnaires under the supervision of trained interviewers. The demographic information included age, educational level, household income, employment status, marital status, cohabiting family composition, and spouse information. Of these, change in marital status and cohabiting family composition were evaluated at each visit.

**Table 2.** Details of data and material collection in KPOS study

Classification	Contents	Methods
Socio-demographics	▪ Age, education, household income, employment, marriage, cohabitation, spouse’s information	Interview-based questionnaires

Medical and family history	▪ Past history of disease (hypertension, diabetes mellitus, thyroid disease, congenital heart disease, asthma, chronic renal disease, autoimmune disease, asthma, epilepsy, chronic hepatitis, tuberculosis, polycystic ovarian disease, depression, other mental illness)	Interview-based questionnaires/ ^a^Medical record
	▪ Family history of disease (hypertension, diabetes mellitus, gestational diabetes mellitus, preeclampsia, depression, other mental illness)	
	▪ Medication treatment history	

Reproductive information	▪ Current pregnancy information (last normal menstrual period, conception method, foetal number, gestational age, estimated date of delivery, planned pregnancy or not, presence of morning sickness, and health supplement intake)	Interview-based questionnaires/ ^a^Medical record
	▪ Past pregnancy information (gravidity, parity, living babies, stillbirths, abortions, gestational age at prior deliveries neonatal birthweights, GDM, hypertensive disorder in pregnancy, and depression during pregnancy and/or postpartum)	
	▪ Results of pregnancy (mother: date and type for end of pregnancy, mode of delivery (vaginal/ Cesarean), blood pressure, anthropometry (height, weight), biological specimens status, and complications at delivery; baby: number of live births, sex, neonatal anthropometry (height, weight, head circumference), presence of malformations, and Apgar scores)	
	▪ Breast feeding	

Health-related behaviours	▪ Cigarette smoking (lifetime smoking, current smoking, duration, and amount)	Interview-based questionnaires
	▪ Alcohol intake (lifetime drinking, current drinking, duration, and amount)	
	▪ Physical activity before and during pregnancy (frequency and duration of walking, moderate- and vigorous-intensity activity)	
	▪ Sleep duration	
	▪ Dietary intake (food frequency questionnaire)	
	▪ Health supplement intake	

Psychological health	▪ Perceived stress (Distress thermometer, 0: not distressed to 10: extremely distressed)	Interview-based questionnaires
	▪ Satisfaction with marriage (0: extremely unhappy to 6: perfectly happy)	
	▪ Depression (Korean version of the Edinburgh Postnatal Depression Scale, ≥10: depression)	
	▪ Quality of life (Korean version of the European Quality of Life 5 Dimension, three levels of response: no, some, and extreme problems)	

Clinical and laboratory measurement	▪ Anthropometry (height, weight)	^a^Medical record
	▪ Blood pressure	
	▪ Blood tests (haemoglobin, haematocrit, glucose, liver enzyme, BUN, creatinine, cholesterol, serology for aneuploidy, 50-g GCT, and 75-g or 100-g OGTT)	
	▪ Urinary tests (glycosuria and proteinuria)	

Pregnancy outcomes	▪ Maternal illness during pregnancy (hyperemesis, threatened abortion, GDM, hypertensive disorders of pregnancy, peripartum depression, and treatment)	^a^Medical record

Biological specimens	▪ Maternal blood and placenta	^a^Medical record

GDM, gestational diabetes mellitus; BUN, blood urea nitrogen; GCT, glucose challenge test; KPOS, Korean Pregnancy Outcome Study; OGTT, oral glucose tolerance test.

^a^Clinically assessed data were transferred from medical record.

#### Medical and familial history

The past medical histories of the participants, including a history of medical diseases and treatments, such as hypertension, diabetes mellitus, thyroid disease, congenital heart disease, asthma, chronic renal disease, asthma, epilepsy, chronic hepatitis, tuberculosis, polycystic ovarian disease, depression, and other mental illness, were determined. The family history data obtained included a family history of hypertension, diabetes, GDM, preeclampsia, depression, and other mental illnesses. These data were evaluated at visit 1.

#### Reproductive information

The basic obstetric information obtained included gravidity, parity, number of living babies, stillbirths, and abortions. The prenatal records of participants were reviewed and data, including the method of conception, number of fetuses, gestational age, estimated date of delivery, and planned pregnancy or not, were extracted. Pregnancy outcomes of past pregnancies, including gestational age at delivery, neonatal birthweight, GDM, hypertensive disorders in pregnancy, and screening positive for peripartum depression during pregnancy and postpartum, were also evaluated.

#### Health-related behaviours

Cigarette smoking, alcohol intake, and supplement intake were evaluated at each visit. Physical activity was assessed at each visit using the short form of the International Physical Activity Questionnaire (IPAQ). Dietary intake patterns were assessed with a specific questionnaire at visit 1.

#### Psychological health

Screening positive for depression during pregnancy and postpartum was evaluated using the Korean version of the Edinburgh Postnatal Depression Scale (K-EPDS), which is a validated questionnaire that includes 10 items.^30^^,^^31^ This scale was specially designed for detecting depression during the postpartum period and was also used to screen for depression during pregnancy.^32^ In addition, we investigated the perceived stress, satisfaction with marriage, and quality of life in this study. Detailed description of survey method were represented in Table 2.

#### Clinical and laboratory measurements in pregnancy

Clinical parameters during pregnancy included height, weight before and after pregnancy, and blood pressure. Laboratory information included haemoglobin, haematocrit, glucose, liver enzyme, blood urea nitrogen (BUN), creatinine, and cholesterol levels, which were recorded at visits 1 and 3. Urinary tests to evaluate for glycosuria and proteinuria were performed at each visit. The results of each serum screening parameter for aneuploidy were recorded at visit 2.

#### Pregnancy outcomes

First trimester complications including hyperemesis and threatened abortion were recorded at visit 1. In patients who had the hyperemesis and threatened abortion, management options were determined by obstetricians according to the degree of the diseases after clinical evaluation. The delivery outcomes included gestational age at delivery, type of labour (induced or spontaneous), type of delivery, indication for Cesarean delivery, and delivery complications. Neonatal outcomes included birthweight, Apgar score at 1 and 5 minutes, height, head circumference, admission to neonatal intensive care unit, intubation, seizure, phototherapy, and presence of congenital anomaly.

#### Pregnancy complications

The main outcomes of this study were GDM, hypertensive disorders of pregnancy, and screening positive of depression during pregnancy and postpartum. GDM was diagnosed using a two-step method. Universal screening with a 50-g glucose challenge test (GCT) was conducted between 24 and 28 weeks. If the result of the GCT was 140 mg/dL or more, a confirmatory oral glucose tolerance test (OGTT) was performed. Two different confirmatory tests were conducted. The Cheil General Hospital used the 75-g OGTT as the confirmatory test and used the new diagnostic criteria from the International Association of Diabetes and Pregnancy Study Group (at least one abnormal value: fasting glucose ≥92 mg/dL, 1-hour glucose ≥180 mg/dL, or 2-hour glucose ≥153 mg/dL). The CHA Gangnam Medical Center used the 100-g OGTT and the Carpenter-Coustan criteria for GDM diagnosis (2 or more abnormal values: fasting glucose ≥95 mg/dL, 1-hour glucose ≥180 mg/dL, 2-hour glucose ≥155 mg/dL, or 3-hour glucose ≥140 mg/dL).^33^^,^^34^ The participants who were diagnosed GDM were sent to endocrinologist, and management methods were determined after clinical evaluation. The information regarding the diagnosis of GDM and insulin therapy were recorded at visit 3 and at delivery.

Blood pressures were measured at every visit using the automatic oscillometric technique, but a diagnosis of hypertensive disorders of pregnancy was confirmed via manual measurements using blood pressure cuffs and auscultation. Blood pressure measurements were taken during visits 2 and 3 and at delivery to identify hypertensive disorders of pregnancy, which were classified into gestational hypertension, preeclampsia, eclampsia, and superimposed preeclampsia.^35^ Gestational hypertension was defined by a systolic blood pressure ≥140 mm Hg and/or diastolic blood pressure ≥90 mm Hg without proteinuria (<0.3 g in a 24-hour urine collection) and the hypertension must have developed after 20 weeks of gestation. Preeclampsia was defined as gestational hypertension plus proteinuria (≥0.3 g in a 24-hour urine collection or ≥1+ on a semi-quantitative dipstick test), or with end-organ dysfunction based on a platelet count <100,000/mm^3^, a creatinine level >1.1 mg/dL, elevation of serum transaminase, pulmonary oedema, or neurologic symptoms, all occurring after 20 weeks of gestation. Eclampsia was defined as preeclampsia with seizures unrelated to other cerebral conditions. The diagnosis of superimposed preeclampsia was based on the development of symptoms of preeclampsia in a pregnant woman with chronic hypertension.

Screening positive for perinatal depression can be applied to the case that current or most recent episode of major depression occurs during pregnancy or in the 4 weeks following delivery. A score of ≥10 on K-EPDS indicated a positive screen for perinatal depression in this study according to previous report.^31^

#### Collection of biological samples

The blood samples (10 mL) were obtained a total of 3 times during pregnancy and once more at 6–8 weeks postpartum in GDM patients only. Three sections of the placenta (1.0 × 1.0 cm^3^) were excised from underneath the membrane as close to the umbilical cord as possible at the time of delivery. The placenta sampling (1.0 cm^2^ × 3) procedure was carried out at the time of delivery. The aim of storing maternal blood and placenta is to identify biomarkers related to pregnancy complications, and to develop prediction tools to improve pregnancy outcomes in the future. Those specimens were stored in −70°C freezers at a controlled temperature and humidity. All biological samples were marked with barcodes and stored in the National Biobank of Korea, which was constructed by the Korea Centers for Disease Control and Prevention. We uploaded the data from all questionnaires and examinations to a web-based clinical data management system (iCReaT) managed by the Korea National Institute of Health. The data were periodically monitored by an independent data management team for continuous quality control.

## RESULTS

The baseline characteristics of the study participants are presented in Table 3. The study included a total of 4,195 pregnancies and the median age of the participants was 33.0 years. Most participants were married (97.0%), and the proportion of those who graduated college or university was 74.5%. The distribution of household income peaked at ≥5 million Korean Won/month (47.6%). The participants’ most common occupation in early pregnancy was a full-time employee (52.6%). More than half of the participants (62.1%) were nulliparas, 33.1% were primiparas, and 4.8% were multiparas. The proportion of women with a histories of miscarriage or abortion was 12.5%, and those with histories of comorbid disorders were as follows: 0.6% had hypertension and diabetes mellitus, 0.7% had depression, 1.7% had polycystic ovarian syndrome, and 6.2% had thyroid hormone dysfunction. Among multiparous women, GDM was the most frequently occurring comorbid condition, affecting 4.2% of the participants, followed by peripartum depression affecting 3.1%, and hypertensive disorders affecting 1.5% of women in a previous pregnancy. Among the 4,194 pregnancies, 0.9%, 1.1%, and 4.1% of the women conceived via ovulation induction, artificial insemination, and *in vitro* fertilization, respectively. Most of the participants had singleton pregnancies (98.4%) in the current pregnancy. Most of them were former drinkers (80.1%), but the prevalence of former smokers was very low (10.9%). The frequencies of current smokers and drinkers were very low (0.1%) since almost all of the pregnant women tried to stop smoking and drinking during their pregnancies. The participants who were underweight (body mass index [BMI] <18.5 kg/m^2^) or overt obesity (BMI ≥30.0 kg/m^2^) before pregnancy accounted for 15.0% and 1.5% of the participants, respectively.

**Table 3.** Baseline characteristics of the study participants

Variables	Number of valid responses	Total (*n* = 4,195)	
Maternal age, years	4,195	33.0	[31.0–36.0]
<20		1	(0.0)
20–24		41	(1.0)
25–29		682	(16.3)
30–34		1,963	(46.8)
35–39		1,273	(30.3)
≥40		235	(5.6)

Marital status			
Married		4,068	(97.0)
Unmarried		121	(2.9)
Divorced/Widowed/Separated		6	(0.1)
Cohabitation			
Single person household		23	(0.5)
Couple only		2,377	(56.7)
Couple with child(ren)		1,321	(31.5)
Other		474	(11.3)
Educational status			
≤High school		361	(8.6)
College or university		3,125	(74.5)
≥Graduate school		709	(16.9)

Household Income, million Korean-Won/month			
<2		93	(2.2)
2–3		451	(10.8)
3–4		748	(17.8)
4–5		907	(21.6)
≥5		1,996	(47.6)
			
Occupation in early pregnancy			
Owner-operator/Employer		272	(6.5)
Full-time employee		2,207	(52.6)
Part-time/Temporal employee		143	(3.4)
Others (Housewife/Students/Unemployed)		1,573	(37.5)
Blood pressure at the first trimester, mm Hg			
Systolic blood pressure		113.0	[105.0–122.0]
Diastolic blood pressure		66.0	[60.0–72.0]
BMI before pregnancy, kg/m^2^		20.7	[19.2–22.5]
<18.5		626	(15.0)
18.5–22.9		2,688	(64.4)
23.0–24.9		442	(10.6)
25.0–29.9		357	(8.6)
≥30.0		62	(1.5)
			
BMI at the first trimester, kg/m^2^		21.1	[19.6–23.1]
<18.5		436	(10.4)
18.5–22.9		2,660	(63.4)
23.0–24.9		561	(13.4)
25.0–29.9		448	(10.7)
≥30.0		90	(2.1)
			
Parity			
0		2,606	(62.1)
1		1,387	(33.1)
≥2		202	(4.8)
History of miscarriage/abortion		525	(12.5)
History of diseases			
Hypertension		27	(0.6)
Diabetes mellitus		26	(0.6)
Depression		31	(0.7)
Polycystic ovarian syndrome		72	(1.7)
Thyroid hormone dysfunction		260	(6.2)
History of pregnancy-related complications	1,589		
Gestational diabetes mellitus		67	(4.2)
Hypertensive disorders of pregnancy		24	(1.5)
Gestational hypertension		11	(0.7)
Preeclampsia/Eclampsia		13	(0.8)
Peripartum depression		49	(3.1)
Depression during pregnancy		8	(0.5)
Postpartum depression		56	(3.5)

Type of pregnancy	4,194		
Normal		3,960	(94.4)
Ovulation induction		36	(0.9)
Artificial insemination		46	(1.1)
In vitro fertilization		172	(4.1)
Cigarette smoking			
Never smoked		3,731	(89.0)
Former smoker		458	(10.9)
Quit before pregnancy		343	(74.9)
Quit after pregnancy		115	(25.1)
Current smoker		5	(0.1)
Passive smoking		1,528	(36.4)
Alcohol consumption			
Never drank		828	(19.7)
Former drinker		3,361	(80.1)
Quit before pregnancy		2,025	(60.2)
Quit after pregnancy		1,336	(39.8)
Current drinker		5	(0.1)

Data expressed as median [interquartile range] or numbers (percentages).

BMI, body mass index.

Of the 4,195 pregnant women recruited into the KPOS, 3,565 (85.0%) pregnancy outcomes were observed in the cohort, including 30 miscarriages, 16 stillbirths, and 3,519 deliveries (Table 4). The proportion of women with a history of hyperemesis and threatened abortion was 76.1% and 18.0%, respectively. The average gestational age at birth was 38.8 ± 1.5 weeks. Of those, 2,097 women (59.6%) delivered vaginally, and 1,422 women (40.4%) delivered via Caesarean section. The proportion of participants who had complications at delivery was 11.5%, and premature rupture of membranes was the most prevalent complication (7.0%). A total of 55.6% of women had excessive or sub-adequate gestational weight gain during pregnancy, based on the Institute of Medicine guidelines.^36^

**Table 4.** Pregnancy results

Variables	Number of valid responses	Total (*n* = 3,565)	
Pregnancy result	3,565		
^a^Miscarriage/abortion		30	(0.8)
^b^Stillbirth		16	(0.4)
Delivery		3,519	(98.7)
Hyperemesis		2,713	(76.1)
Mild		2,056	(75.8)
Severe		635	(23.4)
Inpatient treatment		22	(0.8)
Threatened abortion		641	(18.0)
Stabilization		484	(75.5)
Drug treatment		125	(19.5)
Inpatient treatment		32	(5.0)
Gestational age at birth, weeks	3,519	39.0	[38.0–40.0]
Pre-term (<37)		195	(5.5)
Normal (37–41)		3,322	(94.4)
Post-term (≥42)		2	(0.1)
Type of delivery			
Vaginal		2,097	(59.6)
Cesarean section		1,422	(40.4)
Complications at delivery		404	(11.5)
Injuries of parturient canal		125	(3.6)
Abruption placenta		10	(0.3)
Premature rupture of membranes		248	(7.0)
Other		50	(1.4)
Blood pressure at delivery, mm Hg			
Systolic blood pressure		118.0	[110.0–124.0]
Diastolic blood pressure		72.0	[67.0–80.0]
BMI at delivery, kg/m^2^		25.8	[24.0–28.0]
<18.5		2	(0.1)
18.5–22.9		470	(13.4)
23.0–24.9		862	(24.5)
25.0–29.9		1,739	(49.4)
≥30.0		446	(12.7)
^c^Gestational weight gain			
<Recommended by IOM		1,168	(32.8)
Adequate		1,585	(44.4)
>Recommended by IOM		812	(22.8)

Number of live births	3,519	3,568	(101.4)
Singleton		3,470	(98.6)
Twin		49	(1.4)
Sex	3,568		
Boy		1,838	(51.5)
Girl		1,730	(48.5)
Neonatal anthropometry			
Height, cm		49.8	[48.3–51.0]
Weight, kg		3.2	[3.0–3.5]
Head circumference, cm		34.5	[33.8–35.4]
Apgar score			
1 minute		8.0	[8.0–8.0]
5 minutes		9.0	[9.0–9.0]
Neonatal intensive care unit		423	(11.9)

Data expressed as median [interquartile range] or numbers (percentages).

BMI; body mass index; IOM, Institute of Medicine.

^a^Miscarriage is defined as the spontaneous loss of a foetus at <20 weeks of gestation.

^b^Stillbirth is defined as delivery of a foetus showing no signs of life at ≥20 weeks of gestation.

^c^Adequate gestational weight gain during pregnancy is defined as 12.5–18.0 kg for underweight, 11.5–16.0 kg for normal weight, 7.0–11.5 kg for overweight, and 5.0–9.0 kg for obese women.

Among the 3,519 deliveries, 3,568 live births occurred, in which the infants were more frequently boys (51.5%) and the births included 49 sets of twins. The mean anthropometric measurements for height, weight, and head circumference of the baby were 49.6 cm, 3.2 kg, and 34.5 cm, respectively. The mean Apgar scores were 7.9 and 8.8 at 1 and 5 minutes, respectively.

Table 5 shows the frequency of current pregnancy complications among the study participants who have results in pregnancy. The prevalence rates of pregnancy complications were 7.0% for GDM, 1.4% for hypertensive disorders of pregnancy, 27.8% for screening positive of depression during pregnancy, and 16.6% for screening positive for postpartum depression. Screening positive of depression during pregnancy occurred in 19.5%, 13.7%, and 13.9% of participants in the 1^st^, 2^nd^, and 3^rd^ trimesters, respectively. We’ve additionally present the result of age-specific and parity-specific perinatal pregnancy-related outcomes analyses in eTable 2 and eTable 3, respectively. In eTable 2, we showed the pregnancy complications and perinatal outcomes according to maternal age. As maternal age increases, the prevalence of pregnancy complications tend to be increased. In comparison with nulliparous women, the prevalence of hyperemesis, GDM, screening positive for depression during pregnancy were significantly higher in multiparous women. In contrast, the complications at delivery were significantly more common in nulliparous women (eTable 3).

**Table 5.** Frequency of pregnancy-related complications

Variables	Number of valid responses	Total (*n* = 3,565)	
Gestational diabetes mellitus	3,565	250	(7.0)
Screening positive for gestational diabetes mellitus		867	(24.3)
Hypertensive disorders of pregnancy		49	(1.4)
Gestational hypertension		16	(32.7)
Preeclampsia		32	(65.3)
Eclampsia		1	(2.0)
Screening positive for peripartum depression			
Screening positive of depression during pregnancy		990	(27.8)
1^st^ trimester		790/4,053	(19.5)
2^nd^ trimester		488/3,555	(13.7)
3^rd^ trimester		424/3,050	(13.9)

Screening positive for postpartum depression	2,530	420	(16.6)

Data expressed as numbers (percentages).

## DISCUSSION

To the best of our knowledge, the KPOS is the first large-scale prospective study to focus on the limited scientific understanding of risk factors and the frequency of pregnancy complications in Korea. Data derived from the Korean Statistical Information Service, the average maternal age in 2016 was 32.4 years.^37^ According to the Korea National Health and Nutrition Examination Survey, the percentage of smoking and drinking history among Korean women were 7.5% and 73.6%, separately.^38^ In comparison with Korea national population data, the enrolled patients of KPOS pregnant women are thought to be adequate to represent the Korean general reproductive-aged women (KPOS data: 33.0 years, 10.9%, and 80.3%, respectively). However, socioeconomic variables such as educational status (college or university degree or more: 40.5% vs 91.4%) and household income (3 million Korean-Won or more: 51.1% vs 87.0%) were different with Korea national population data, presumably because both hospitals are located in the center of Seoul city.^37^^–^^39^

The prevalence of pregnancy-related complications have increased as the number of women with advanced maternal age continues to grow.^11^^,^^13^^,^^18^^,^^40^^–^^44^ An increasing trend in GDM prevalence has been reported globally.^41^^–^^44^ In a previous meta-analysis, the pooled prevalence of GDM in Eastern and South-eastern Asia was 10.1%, and more specifically, a 64.0% higher prevalence was observed among lower or upper-middle income countries.^43^ According to data from the Korean National Health Insurance Review and Assessment database, the prevalence of GDM significantly increased from 5.7% in 2009 to 9.5% in 2011.^42^ Similar to a previous Korean study, the prevalence of GDM was 7.0% in the present study. In contrast, this study found a very low prevalence (1.4%) of hypertensive disorders of pregnancy, compared to the prevalence of other countries. Among the 3,565 participants, 16, 32, and 1 of the participants developed gestational hypertension, preeclampsia, and eclampsia, respectively, during pregnancy. A previous study from the Korean National Health Insurance Service–National Sample Cohort reported that the overall prevalence rates of hypertensive disorders of pregnancy requiring magnesium sulfate therapy were 5.7, 4.7, and 4.1 per 1,000 pregnant women for gestational hypertension, preeclampsia, and eclampsia, respectively.^40^ Epidemiologic studies showed that the most cited and accepted estimate of the occurrence of hypertensive disorders of pregnancy is 5–10%, and the prevalences of hypertensive disorders of pregnancy, gestational hypertension, and preeclampsia are 5.2–8.2%, 1.8–4.4%, and 0.2–9.2%, respectively.^45^^,^^46^ KPOS participants were also more likely to be well educated, with higher income, were more frequently full-time employee, had relatively lower BMIs, and had healthy lifestyle factors, all reflective of the characteristics of the urban middle class in Korea. High socioeconomic status and appropriate prenatal care among the study participants may have led to the relatively low incidence of hypertensive disorders of pregnancy in this study compared to that of other studies.

Depression is a common complication of pregnancy and the postpartum period. Recent meta-analysis suggested that the incidence of postpartum depression was 12.0%, while the prevalence was 17.0%.^47^ There were differences between different geographical regions; however, the prevalence rates were similar, regardless of the type of diagnostic tool used. The prevalence of screening positive of depression at least once during pregnancy was 27.8%, and the prevalence of having screened positive for peripartum depression was 16.6%. A previous epidemiologic study in Korea showed that the prevalence of peripartum depression using the K-EPDS was found to be 18.8% in the 1st trimester, 12.9% in the 2nd trimester, 12.6% in the 3rd trimester, and 15.7% at 1 month after delivery.^48^ This was very similar with our results, and psychiatric treatment would be recommended to help depressed pregnant women.

The main strengths of the KPOS include the following: 1) a prospective design that aims to understand key determinants of adverse pregnant outcomes to assist in determining causal relationships between pregnancy exposures and complications; 2) we have collected abundant information from the interview-based questionnaires, biological specimens, and clinical data obtained at multiple time points during the pregnancies to assess the independent and interactive effects of various factors on pregnancy complications; and 3) our KPOS investigate team has provided optimal obstetric care to the participants at Cheil General Hospital and CHA Gangnam Medical Center, both well-known maternity hospitals in Korea, inclusive of both low- and high-risk pregnant women.

There are some limitations to be considered in this study. First, our study population may not have been representative of all Korean pregnant women because the socioeconomic status of our study participants tend to be higher than those of Korean national population; thus, the results need to be interpreted with cautious. Second, some of the KPOS data were collected via maternal self-report, which could have led to potential bias. Particularly with respect to cigarette smoking and alcohol consumption during pregnancy, self-reports of substance use may have underestimated actual use due to the negative connotations and stigmatization. Third, despite the quite large sample size of the study population, our data lacked the power to investigate relatively rare outcomes, such as preeclampsia or eclampsia, in Korea. Fourth, the extent of detailed and comprehensive questionnaires conducted in each trimester may have reduced the follow-up rate. Finally, the follow-up duration was relatively short because long-term follow-up was practically limited as pregnant women after delivery were scattered into each community. Although time was short to observe the transition from pregnancy complications to chronic diseases, it is expected that research using KPOS data will be actively conducted in the future.

### Conclusions

Given the increasing trend of advanced maternal age in Korea, we designed KPOS to provide evidence for preventing pregnancy complications. An understanding of the pregnancy-associated risk indicators may help to identify women who are at increased risk of pregnancy-related adverse outcomes later in life. Future studies on the KPOS data will provide information on the determinants of pregnancy-related disorders among pregnant women in Korea. The results will contribute to management of pregnancy-related complications, and thus improve overall maternal and neonatal health.

## APPENDIX A. SUPPLEMENTARY DATA

eTable 1. Sample size calculation in KPOS

eTable 2. Age-specific perinatal pregnancy-related complications

eTable 3. Parity-specific perinatal pregnancy-related complications
