# Supplementary material for: The Korean Pregnancy Outcome Study (KPOS): Study Design and Participants
Source: J Epidemiol. 2021 Jun 5;31(6):392–400. doi: 10.2188/jea.JE20200055 (PMC8126675; doi:10.2188/jea.JE20200055)
Supplement: Supplementary file 1 [file je-31-392-s001.pdf]

eTable 1. Sample size calculation in KPOS

Sample size	Cumulative incidence	Minimum detectable odds ratio when comparing		
		High 33% vs. Low 67%	High 25% vs. Low 75%	High 25% vs. Low 25%
3,000	5%	1.64	1.70	1.91
	10%	1.45	1.49	1.64

Two-tailed test; α=0.05; β=0.2; Exposure correlation coefficient=0.2;

eTable 2. Age-specific perinatal pregnancy-related complications

Variables	No. of valid response	Age, years old ≤30	31–35	36–40	>40	*p*-value
Pregnancy result	***3,565***	***832 (23.3)***	***1,741 (48.8)***	***880 (24.7)***	***112 (3.1)***	
[a] Miscarriage / abortion		7 (0.8)	13 (0.7)	9 (1.0)	1 (0.9)	
[b] Stillbirth		1 (0.1)	9 (0.5)	4 (0.5)	2 (1.8)	0.177
Delivery		824 (99.0)	1,719 (98.7)	867 (98.5)	109 (97.3)	
Emesis		615 (73.9)	1,329 (76.3)	678 (77.0)	91 (81.3)	0.231
Threatened abortion		150 (18.0)	286 (16.4)	178 (20.2)	27 (24.1)	0.033
Gestational diabetes mellitus		32 (3.8)	118 (6.8)	87 (9.9)	13 (11.6)	<0.001
Hypertensive disorders of pregnancy		9 (1.1)	20 (1.1)	19 (2.2)	1 (0.9)	0.175
Gestational hypertension		4 (44.4)	8 (40.0)	4 (21.1)	0 (0.0)	
Preeclampsia		4 (44.4)	12 (60.0)	15 (78.9)	1 (100.0)	0.419
Eclampsia		1 (11.1)	0 (0.0)	0 (0.0)	0 (0.0)	
Screening positive for depression during pregnancy		197 (23.7)	495 (28.4)	264 (30.0)	34 (30.4)	0.020
Screening positive for postpartum depression	***2,530***	84 (14.4)	210 (16.8)	105 (17.3)	21 (24.1)	0.054
Gestational age at birth, weeks	***3,519***	***824 (23.4)***	***1,719 (48.8)***	***867 (24.6)***	***109 (3.1)***	
Pre-term (<37)		38 (4.6)	89 (5.2)	61 (7.0)	7 (6.4)	
Normal (37–41)		784 (95.1)	1,630 (94.8)	806 (93.0)	102 (93.6)	0.058
Post-term (≥42)		2 (0.2)	0 (0.0)	0 (0.0)	0 (0.0)	
Complications at delivery		98 (11.9)	173 (10.1)	114 (13.1)	19 (17.4)	0.021

Data expressed as numbers (percentages)

[a] Miscarriage is defined as the spontaneous loss of a foetus at <20 weeks of gestation.

[b] Stillbirth is defined as delivery of a foetus showing no signs of life at ≥20 weeks of gestation.

eTable 3. Parity-specific perinatal pregnancy-related complications

Variables	No. of valid response	Parity Nullipara		Multipara		*p*-value
Pregnancy result	***3,565***	***2,130***	***(59.7)***	***1,435***	***(40.3)***	
[a] Miscarriage / abortion		16	(0.8)	14	(1.0)	
[b] Stillbirth		10	(0.5)	6	(0.4)	0.897
Delivery		2,104	(98.8)	1,415	(98.6)	
Emesis		1,544	(72.5)	1,169	(81.5)	<0.001
Threatened abortion		397	(18.6)	244	(17.0)	0.230
Gestational diabetes mellitus		126	(5.9)	124	(8.6)	0.002
Hypertensive disorders of pregnancy		33	(1.5)	16	(1.1)	0.249
Gestational hypertension		9	(27.3)	7	(43.8)	
Preeclampsia		23	(69.7)	9	(56.3)	0.537
Eclampsia		1	(3.0)	0	(0.0)	
Screening positive for depression during pregnancy		548	(25.7)	442	(30.8)	0.001
Screening positive for postpartum depression	***2,530***	258	(16.7)	162	(16.4)	0.827
Gestational age at birth, weeks	***3,519***	***2,104***	***(59.8)***	***1,415***	***(40.2)***	
Pre-term (<37)		116	(5.5)	79	(5.6)	
Normal (37–41)		1,989	(94.5)	1,336	(94.4)	0.509
Post-term (≥42)		2	(0.1)	0	(0.0)	
Complications at delivery		309	(8.8)	95	(2.7)	<0.001

Data expressed as numbers (percentages)

[a] Miscarriage is defined as the spontaneous loss of a foetus at <20 weeks of gestation.

[b] Stillbirth is defined as delivery of a foetus showing no signs of life at ≥20 weeks of gestation.
